# Infectious or Noninfectious? Ruptured, Thrombosed Inflammatory Aortic Aneurysm with Spondylolysis

**DOI:** 10.1007/s00270-012-0464-3

**Published:** 2012-09-13

**Authors:** Ludomir Stefańczyk, Marcin Elgalal, Andrzej Papiewski, Wojciech Szubert, Piotr Szopiński

**Affiliations:** 1 Department of Radiology and Diagnostic Imaging, Medical University of Łódź, ul. Kopcińskiego 22, 90-159 Łódź, Poland; 2 Department of Gastroenterological Surgery, Medical University of Łódź, ul. Kopcińskiego 22, 90-159 Łódź, Poland; 3 Clinic of Vascular Surgery, Institute of Hematology and Transfusion Medicine, Indira Gandhi 14 St, Warsaw, 02-776 Poland

**Keywords:** Abdominal aortic aneurysm, Inflammatory aneurysm, Stent graft, Vertebral destruction

## Abstract

Osteolysis of vertebrae due to inflammatory aortic aneurysm is rarely observed. However, it is estimated that up to 10 % of infectious aneurysms coexist with bone tissue destruction, most commonly the vertebrae. Inflammatory aneurysms with no identified infection factor, along with infiltration of adjacent muscle and in particular extensive destruction of bone tissue have rarely been described in the literature. A case of inflammatory aneurysm with posterior wall rupture and inflammatory infiltration of the iliopsoas muscle and spine, together with extensive vertebral body destruction, is presented. The aneurysm was successfully treated with endovascular aneurysm repair EVAR.

## Introduction

Osteolysis of vertebrae due to inflammatory aortic aneurysm is rarely observed [1–4]. However, it is estimated that up to 10 % of infectious aneurysms coexist with bone tissue destruction, most commonly the vertebrae [5]. In atherosclerotic aneurysms, shallow erosions of neighboring vertebral bodies, which develop as a result of chronic compression, can be observed in 0.6 % of cases [6]. The presence of an inflammatory aneurysm with no identified infection factor, as well as infiltration of the iliopsoas muscle and in particular extensive destruction of adjacent vertebrae, has rarely been described in the literature [7, 8]. A case of inflammatory aneurysm with posterior wall rupture and inflammatory infiltration of the iliopsoas muscle and spine, together with extensive vertebral body destruction, is presented. The aneurysm was successfully treated by implantation of a bifurcated stent graft.

## Case Report

**Fig. 1:**
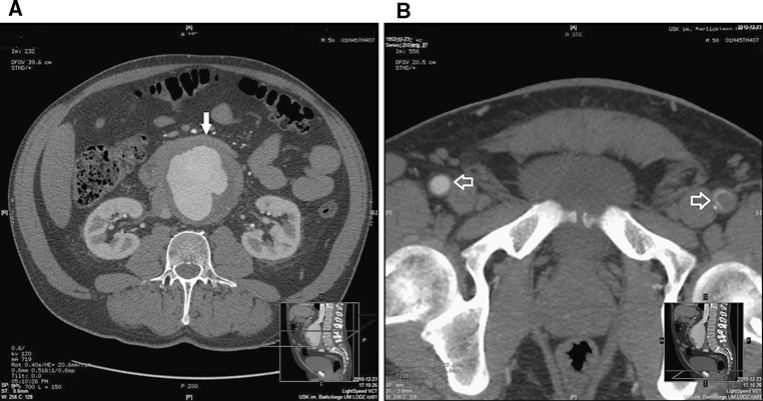
Angio-CT axial scan, aortic aneurysm below the level of the renal arteries, with irregular adhering thrombus. **A** Within the anterior aortic wall there is a narrow, irregular soft tissue infiltration that suggests the presence of inflammation (mantle core sign) (*arrow*). **B** Axial scan at the level of femoral arteries (*arrows*) reveals an embolism of the left artery. The right femoral artery is patent

A 59-year-old man was admitted to the vascular surgery department as a result of acute ischemia of the lower limb in December 2010. The patient also complained of weakness, lack of appetite, and weight loss that had developed in the preceding several months (20 kg in the past 4 months). He had a several-week history of abdominal pain with radiation to the lumbar region, which had recently become exacerbated. The patient did not exhibit voiding dysfunction (urine or stool). Palpation revealed abdominal tenderness in the periumbilical region, with no peritoneal signs. During hospitalization, laboratory test results indicated the presence of an inflammatory process: mild leukocytosis (white blood cells 8,300–10,100/μl), greatly elevated C-reactive protein (CRP)—up to 46 mg/l—and extremely elevated D-dimer levels (6,370–5,920 mg/ml). Ultrasound examination was performed, which revealed an inflammatory aortic aneurysm (diameter >90 mm) and embolism of the left femoral and left popliteal arteries. Computed tomographic (CT) examination (64 × 0.625 mm GE Lightspeed VCT; GE Healthcare, Waukesha, WI) revealed retroperitoneal inflammatory infiltration surrounding the aorta, an abdominal aortic aneurysm 95 mm in diameter situated below the level of the renal arteries, and embolism of the left femoral artery (Fig. 1). A decision was made to perform surgical embolectomy and endovascular intervention (EVAR). The patient provided written informed consent.

During the first stage of treatment, a left-sided embolectomy was performed with a Fogarty catheter, and emboli from the femoral and popliteal arteries were removed. In the 24 h after this procedure, a further embolism of right femoral artery developed, which was removed during a second embolectomy. The next day, an EVAR procedure was carried out in the angiography suite under fluoroscopic control (Axiom-Artis; Siemens, Erlangen, Germany). It was performed with the patient in the supine position and under general anesthesia. Arterial access was achieved via an open femoral arteriotomy. Next, after open exposure and puncture of the right femoral artery, a Lunderquist Extra Stiff Wire Guide, 0.035 inches, 260 cm (Cook, Bloomington, IN), and a stiff guide wire, 0.035 inches, 260 cm (Terumo, Somerset, NJ), were inserted and passed into the aorta. A 5F pigtail was also passed into the aorta via left femoral access and was used to perform angiographies. A stent graft was passed into the aorta via the right femoral artery and placed across the neck of the aneurysm, covering it entirely while preserving flow in the common iliac arteries. The stent grafts used were Zenith: proximal body 28 × 111 mm, and distal branches 18 × 56 and 16 × 73 mm (Cook, Bloomington, IN). A check angiogram revealed no leaking into the aneurysm and maintenance of flow in the iliac arteries. At the start of the procedure, a bolus of 2500 U of heparin was administered, and a similar dose was provided in a continuous saline flush. The patient received prophylactic antibiotic therapy to prevent infection of the stent graft (spiramycin 3 million IU, two tablets per day; meloxicam 15 mg, two tablets per day, for a total of 20 days).

**Fig. 2:**
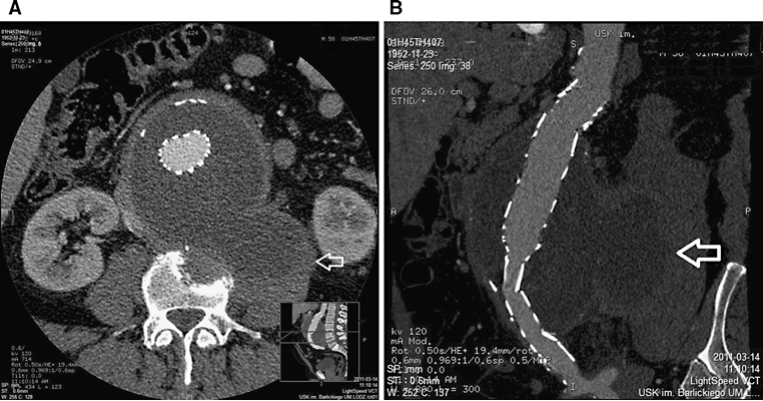
Angio-CT axial scan of aortic aneurysm below renal arteries. After implantation of the stent graft, patency is maintained. **A** Within the anterior and lateral walls neighboring the aorta, there is a soft tissue infiltration encompassing the inferior vena cava. A hematoma and inflammatory infiltration is probably present within the left iliopsoas muscle (*arrow*). Osteolysis of the L4 vertebral body is evident. **B** Reconstruction in the longitudinal plane confirms patency of the graft without leaking into the aneurysmal sac. The hematoma and inflammatory process have infiltrated the area of the iliopsoas muscle (*arrow*)

The patient was discharged in good general condition, with persisting mild pain in the lumbar area, which gradually increased with time. A follow-up angio-CT performed after the procedure in March 2011 confirmed the patency of the implant and confirmed that the aneurysmal sac had been successfully excluded. However, the hematoma and inflammatory infiltration that had been present previously remained visible in the surrounding tissues, with significant progression; it now encompassed the iliopsoas muscle and L4 vertebra with destruction of its body (Fig. 2). Laboratory tests revealed persistent mild leukocytosis and elevated CRP levels (white blood cells 7.500–8.000/μl, CRP 103 mg/l, D-dimer 1,720 mg/ml). Additional hematoma and/or inflammatory infiltration was suspected, and the patient was readmitted to the hospital. An ultrasound-guided biopsy of the described lesion in the iliopsoas muscle was performed (Vivid 7; GE, Waukesha, WI). The presence of pus was not confirmed, and there was no bacterial growth from the aspirated material. Cytological examination revealed the presence of inflammatory granulation tissue (macrophages, lymphocytes, numerous stimulated mesothelial cells). Blood culture and tuberculosis test were both negative. Antibiotic and anti-inflammatory treatment were administered (clindamycin 3 × 300 mg, metronidazole 2 × 250 mg, omeprazole 2 × 1 tablet).

**Fig. 3:**
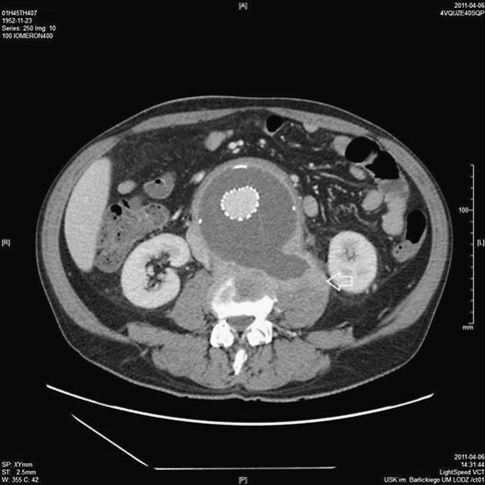
Angio-CT axial scan of aneurysm below renal arteries after EVAR. Soft tissue infiltration with postcontrast enhancement is visible, which encompasses the aorta, the inferior vena cava, and the hematoma in the left iliopsoas muscle (*arrow*). Osteolysis of the L4 vertebral body is evident

**Fig. 4:**
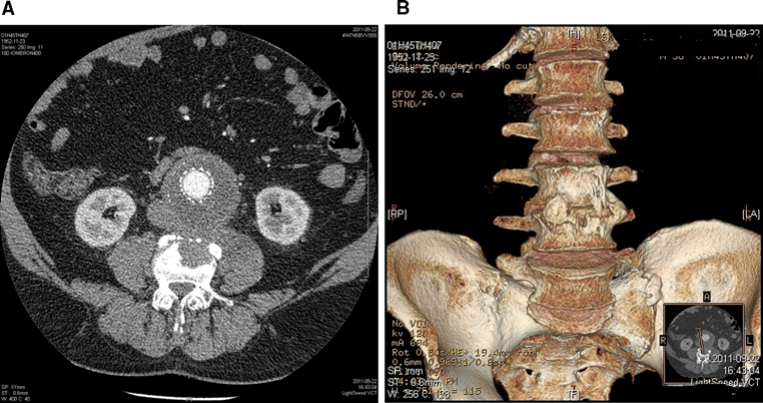
Angio-CT axial scan of aneurysm below renal arteries after EVAR. **A** Regression of the inflammatory changes in the vicinity of the aorta and iliopsoas muscle is evident. Visible bone remodeling and new bone formation of damaged L4 body are present. **B** 3-D reconstruction of the lumbar spine reveals a proliferative reaction at the level of the affected vertebrae

Follow-up ultrasound and CT in April 2011 revealed a persistent area of inflammation in the iliopsoas muscle and destruction of neighboring vertebral bodies (L3, L4) (Fig. 3); therefore, the patient was rehospitalized. A repeat ultrasound-guided biopsy of the lesion within the iliopsoas muscle was performed. This revealed the presence of inflammatory tissue with no signs of infection (bacterial and mycological tests were once again negative). Blood culture and tuberculosis tests were also negative. White blood cell level was 5.600/μl, CRP 51 mg/l, and D-dimer 2,018–1,732 mg/ml. On the basis of these findings, a final diagnosis of inflammatory aneurysm was established. The spine was stabilized with a Jewette corset, and the patient received steroid treatment (dexamethasone 1 mg/day). During the next few days, the patient’s condition greatly improved, and pain symptoms subsided. Angio-CT was performed 5 months later in September 2011, which revealed patency of the stent graft with no signs of leaking, and the aneurismal sac had become markedly reduced in size. The extent of the retroperitoneal inflammation had diminished, and the affected vertebrae exhibited signs of remodeling and new bone formation (Fig. 4).

## Discussion

This case study is an example of the vertebral bone tissue destruction that can be caused by an inflammatory abdominal aortic aneurysm.

The subject of inflammatory aneurysms and their etiology has been widely discussed [8–10]. It is assumed that aneurysms for which an infection factor cannot be established develop either as a consequence of an autoaggressive process that results in the formation of inflammatory granulation tissue and leads to degenerative changes within the walls of the aorta; or that the inflammatory process associated with the atherosclerotic and degenerative changes within the walls of the aorta spreads to the surrounding tissues [8, 9, 11]. On the other hand, inflammatory aneurysms could develop as a result of infection and inflammation that encompass the thrombus in the aneurysmal sac; alternatively, such a process could cause destruction and rupture of the aortic walls, thus resulting in vessel dilatation [1, 3, 5]. As a result of this, infectious aneurysms are saccular in shape, with characteristic rapid growth and a strong tendency to rupture. They can be localized in any section of the aorta, with infiltration of the neighboring tissues, including the spine [1, 2, 4, 5]. Bone tissue destruction has been described in 5–10 % of cases of infectious aneurysms [3]. In contrast, inflammatory aneurysms without infection are usually fusiform, they are located in the abdominal part of the aorta below the level of the renal vessels, and they tend to rupture less often [3]. Inflammatory infiltration, when present, usually spreads along the anterior and lateral walls of the aorta to the iliac vessels and encompasses the ureters and veins (inferior vena cava and iliac). However, such infiltration usually remains separate from the posterior aortic wall and the spine [5, 8, 9, 12].

Postcontrast CT is the most important diagnostic imaging technique in such conditions [13], and in this particular case, the mantle core sign can be observed [11]. Postcontrast CT imaging also reveals the presence of infiltration (enhanced) in tissues surrounding the aneurysm, which contrasts with the adhering intraluminal thrombus that does not demonstrate any signs of enhancement in the late postcontrast phase [8, 9]. In the presented case, the first images suggested a noninfectious inflammatory aneurysm. The next two CT scans revealed a hematoma within a skeletal muscle, destruction of a vertebrae, and annular enhancement, which were highly suggestive of infectious aneurysm [3, 5, 7]. In the literature, an inflammatory aneurysm diagnosed on the basis of CT imaging, with no established infection factor and infiltration of the iliopsoas muscle, has been previously described; however, in that case, there was no damage to surrounding vertebrae [7]. In the present case, the CT images are similar to those of a case described in the literature of an infected retroperitoneal hematoma observed 2 years after aortic aneurysm rupture [6]. In both types of aneurysm (infectious and noninfectious), laboratory tests revealed changes that indicated the presence of an inflammatory process. Therefore, it is vital to have a positive bacteriological test to differentiate between the two conditions. It is worth noting that in approximately 25 % of cases with morphological and clinical characteristics of infection, no source of infection can be identified. Furthermore, more than 40 % of blood cultures are negative in confirmed cases of infectious aneurysms [3, 4, 12]. In the presented case, it is also worth noting that the level of CRP was high while leukocytosis was relatively low. This strongly indicates a lack of infection. However, the diagnosis of a sterile inflammatory aneurysm was only established after two negative biopsy findings and a negative blood culture. Determining the etiology of the aneurysm is vital when deciding on an effective course of treatment.

In cases of inflammatory aneurysms with no infection, conservative treatment is possible (steroids or immunosuppressive drugs, which are contraindicated in the case of infection). Steroids can reduce the degree inflammation and the diameter of an aneurysm and therefore decrease the risk of rupture [11]. Open surgery or endovascular treatment such as EVAR are also possible [8, 12, 14, 15]. In patients who were treated with EVAR only, the degree of inflammation was also diminished [12, 14]. Despite the controversy surrounding the implantation of stent grafts in areas of inflammation, the results of EVAR treatment of inflammatory aneurysms are promising and are comparable to open surgical intervention [8, 11, 12]. The data in the earliest literature suggest that endovascular treatment of inflammatory aneurysms combined with steroid therapy is most successful [8]. In cases of infectious aneurysms, ligation of an affected artery is advisable, as well as an extra-anatomic bypass to prevent infection of the graft [1, 4, 6]. Additionally, antibiotic treatment consistent with the infection factor should be provided [5]. In general, inflammatory aneurysms rupture less commonly than atherosclerotic aneurysms and even less often when compared to infectious aneurysms [3, 11, 12].

The presence of a thrombus in the aneurysmal sac and its rupture into the iliopsoas muscle resulted in equivocal imaging studies that were highly suggestive of an infectious aneurysm. Despite the ambiguous images, the results of laboratory tests made it possible to commence steroid treatment. The clinical course and follow-up diagnostic imaging confirmed that the established diagnosis of noninfectious inflammatory aneurysm was correct.
